# Dr. Edward Charles Bury

**Published:** 1910-05-28

**Authors:** 


We have to announce the death at Wisbech of Dr. Edward Charles Bury. He studied at St. Andrews University, where he graduated M.D. in 1862, becoming an M.R.C.S. (Eng.) and L.S.A. in the same year. His hospital was University College, and among his appointments were those of hon. consulting surgeon to the North Cambridgeshire Hospital and surgeon to the Wisbech Hospital.

